# The Crosstalk of Adipose-Derived Stem Cells (ADSC), Oxidative Stress, and Inflammation in Protective and Adaptive Responses

**DOI:** 10.3390/ijms21239262

**Published:** 2020-12-04

**Authors:** Luc Rochette, Loubna Mazini, Gabriel Malka, Marianne Zeller, Yves Cottin, Catherine Vergely

**Affiliations:** 1 Equipe d’Accueil (EA 7460), Physiopathologie et Epidémiologie Cérébro-Cardiovasculaires (PEC2), Faculté des Sciences de Santé, Université de Bourgogne, Franche Comté, 7 Bd Jeanne d’Arc, 21000 Dijon, France; marianne.zeller@u-bourgogne.fr (M.Z.); yves.cottin@chu-dijon.fr (Y.C.); cvergely@u-bourgogne.fr (C.V.); 2 Laboratoire Cellules Souches et Régénération Cellulaire et Tissulaire, Centre Interface Applications Médicales (CIAM), Université Mohammed VI Polytechnique, Ben-Guerir 43 150, Morocco; Loubna.MAZINI@um6p.ma (L.M.); gabriel.malka@um6p.ma (G.M.); 3 Cardiology Unit, CHU Dijon Bourgogne, 21000 Dijon, France

**Keywords:** stem cells, oxidative stress, adipose derived stem cells, tissue protection

## Abstract

The potential use of stem cell-based therapies for the repair and regeneration of various tissues and organs is a major goal in repair medicine. Stem cells are classified by their potential to differentiate into functional cells. Compared with other sources, adipose-derived stem cells (ADSCs) have the advantage of being abundant and easy to obtain. ADSCs are considered to be tools for replacing, repairing, and regenerating dead or damaged cells. The capacity of ADSCs to maintain their properties depends on the balance of complex signals in their microenvironment. Their properties and the associated outcomes are in part regulated by reactive oxygen species, which mediate the oxidation-reduction state of cells as a secondary messenger. ADSC therapy has demonstrated beneficial effects, suggesting that secreted factors may provide protection. There is evidence that ADSCs secrete a number of cytokines, growth factors, and antioxidant factors into their microenvironment, thus regulating intracellular signaling pathways in neighboring cells. In this review, we introduce the roles of ADSCs in the protection of cells by modulating inflammation and immunity, and we develop their potential therapeutic properties.

## 1. Introduction

One major goal in repair medicine is the development of stem-cell-based therapies for tissue renewal. As the body ages, there is a regression in the capacity of various tissues to self-renew. Tissue homeostasis and regeneration depend on resident progenitor cell function. Aging-signaling pathways are associated with mitochondrial dysfunction and an increased generation of reactive oxygen species (ROS), limiting stem cell proficiency [1]. Stem cells are classified according to their potential to differentiate into various functional cells. Pluripotent stem cells (PSCs), including embryonic stem cells (ESCs) and induced pluripotent stem cells (iPSCs), have the unique features of self-renewal and pluripotency, and they can differentiate into any type of cell in the human body. The molecular mechanisms that control energy metabolism in PSCs and the modifications that occur during differentiation or reprogramming have already been demonstrated [2]. PSCs have valuable applications in regenerative clinical approaches, but stem-cell-based therapies are still restricted in clinical situations by the limits of genetic manipulation and ethical considerations [3]. In bone marrow, mesenchymal stem cells (MSCs) are localized near the sinusoidal endothelium in association with the resident hematopoietic stem cells (HSCs) [4]. The problem with the use of MSCs is that their differentiation potential, their number, and their maximum life span significantly decrease with age [5]. The development of protocols for both the isolation and characterization of MSCs, including standardized functional analyses for estimating of their biological potential, is complex. Therefore, it is essential to obtain uniform methods for isolating and characterizing MSCs [6]. Over the past decades, several clinical trials have studied the safety, feasibility, and functional role of cell therapies in human patients using specific stem cells: adipose tissue-derived stem cells (ADSCs). In this review, we discuss the defined roles of ADSCs. They play a key role in the protection of cells by modulating inflammation and immunity. The therapeutic properties of ADSCs could be used to the benefit of patients suffering from cardiovascular and muscular diseases, and they are a promising source of future cell-based therapies for tissue regeneration.

## 2. Characteristics of Adipose Tissue-Derived Stem Cells and Implications for Tissue Regeneration

### 2.1. Overall Properties

Compared with other sources, ADSCs have an advantage of being abundant and easy to obtain. A number of different terminologies have been used for ADSCs: processed lipoaspirates, adipose-derived adult stem cells, adipose mesenchymal stem cells, adipose-derived stromal/stem cells, and adipose stromal cells [7].

ADSCs are found abundantly and can be harvested with a minimally invasive procedure, and they can differentiate into multiple cell lineages, including endothelial cells (ECs), cardiomyocytes, smooth muscle cells, hepatocytes, adipocytes, osteocytes, and neurons, in a reproducible manner [8]. The production of paracrine factors by human ADSCs promotes angiogenesis by producing vascular endothelial growth factor (VEGF) and transforming growth factor beta (TGF-β) [9].

ADSCs are considered to be a tool for replacing, repairing, and regenerating dead or damaged cells. ADSCs induce tissue reconstitution, thus correcting tissue defects and improving skin regeneration, and they help with wound healing, thereby promoting skin repair [10].

The therapeutic properties of ADSCs could be used to the benefit of patients suffering from cardiovascular and muscular diseases. Satellite cells are responsible for postnatal growth and the ability of adult skeletal muscle to regenerate. On the contrary, because of the limited regenerative capacity of cardiomyocytes, adult cardiovascular tissue is unable to repair itself or to self-renew after injury. In this context, regenerative therapies are in high demand as a new treatment strategy. Several cell-based strategies are being explored for cardiac regeneration. They are classified under two categories: (1) those aiming to directly remuscularize myocardial scarring or (2) those targeting endogenous mechanisms of repair. The progression of stem cell therapy to early clinical trials has reinforced their role in improving cardiac structure and function [11].

Little is known about the differences in ADSC function and differentiation potential that result from their origins in adipose tissue [12]. Recent studies found that cardiac ADSCs have a greater capacity to differentiate into cardiovascular lineage cells (i.e., cardiomyocytes, endothelial cells, and vascular smooth muscle cells) compared with stem cells derived from other adipose tissue. That is a therapeutically approach using ADSCs for cardiac regeneration in ischemic myocardial diseases. In this area, direct cardiac reprogramming occurred as a novel technology to regenerate damaged myocardium by directly converting endogenous cardiac fibroblasts into induced cardiomyocyte-like cells to renovate cardiac function. Fibroblasts remain a central cell type with regard to post-infarction repair and remodeling. ADSC-conditioned media promotes fibroblast proliferation, suggesting the paracrine activation of fibroblasts by ADSCs [12].

In one study, ADSC-rich cells were isolated from subcutaneous, visceral, cardiac, and subscapular adipose tissue in mice. Then, the in vitro differentiation capacity and therapeutic potential of ADSCs were examined in ischemic myocardium using a mouse model of myocardial infarction. The systemic transfusion of cardiac-derived ADSCs exhibited the highest recovery of cardiac function after myocardial infarction [13].

### 2.2. Low Levels of ROS Production Exhibit Positive Effects on ADSCs

The properties and outcomes of stem cells are in part regulated by ROS, mediating the oxidation-reduction (REDOX) state of cells. The ability of stem cells to maintain their properties depends on the balance of complex signals in their microenvironment. The stem cell niche is a new concept that designates an anatomical compartment. They receive and mediate messages from the periphery about the necessity for tissue repair following stress or injury [14].

One of the stem cell niches that has been the most clearly characterized is that of the HSC and cancer stem cells (CSCs). CSCs exist within tumors and the hypoxic microenvironments that are implicated in the aberrant vasculature of tumor tissue [15]. ADSCs appear to have a significant role in cancer relative to the promotion of tumor development and progression and the process of decline [16].

There is growing interest in the association between the tumor microenvironment and CSCs. Iron homeostasis plays a major role in the tumor microenvironment, and both iron deficiency and iron excess can be detrimental to cellular and organ function due to cell growth arrest and death. In this context, the deleterious process is due to an increase in ROS and oxidative stress (OS). It is thought that a balance between “free” iron availability, ferritin levels, and ROS production controls cell death [17,18]. Iron chelators inhibit the formation of tumor spheres in several types of cancers. Recent data obtained in an in vitro model of neuropathy suggest that an iron chelator such as deferoxamine (DFX) can improve the therapeutic potential of ADSCs. “Preconditioning” with DFX increased the expression of potent neuroprotective factors and cytokines such as interleukins (ILs) IL-4 and IL-5, which have anti-inflammatory activity [19].

In tissues and in several types of cells such as adipocytes and ADSCs, it is likely that both iron homeostasis and energy regulation play a key role in the pathogenesis of certain conditions. However, their specific roles are not completely elucidated. For example, there has been recent interest in the association between obesity, inflammation, and adipocyte iron overload. Lipid peroxidation is an end product of ROS in adipocytes and is iron dependent; iron induces lipolysis through a pro-oxidant mechanism. Iron homeostasis is regulated through the actions of modulators such as hepcidin [20], which acts as an antimicrobial peptide and plays an important role in the clearance of pathogens. Hepcidin and other antimicrobial peptides such as LL-37 are secreted by MSCs [21]. LL-37 treatment appears to enhance the proliferation and migration of human ADSCs expressing formyl peptide receptor like-1 and to increase the secretion of regenerative factors. However, the relationship between the function of LL-37 and ADSCs might depend on the microenvironment in the human body [22].

### 2.3. Relationships between ROS Generation, Inflammation, and the Functions of ADSCs

A number of vascular effects have been associated with the expression of vascular cell adhesion molecules on endothelial cells, although the primary role appears to be the regulation of inflammation. Leukocytes are recruited locally at the site of inflammation in a series of adhesive steps that permit them to attach to the vessel wall and to cross the endothelium. Adhesion molecules are proteins that can be induced by a number of mechanisms including elevated fluid shear stress, inflammation, and ROS. Abnormal endothelial function further promotes a series of inflammatory responses. In this context, ADSC are able to act on vascular inflammatory responses and endothelial dysfunction. The relationship between ADSC function and adaptation to pO_2_ in the vascular microenvironment remains ill-defined. While excess ROS are involved in the process of apoptosis, low levels of ROS in the cells activate receptor types and signaling pathways that influence proliferation. The literature demonstrates that the functional properties of ADSCs are under redox control [23]. ADSCs are responsive to several stimuli, but the primary factor is oxygen tension. A low oxygen level (2–8% O_2_) is a key aspect of the cell niche. Absolute oxygen tension within adipose tissue is very low, and ADSCs exist in low oxygen conditions in the body [14].

The capacity to regenerate some tissues is shared by several organisms. In this field, the determination of cell fate and the maintenance of MSCs in an undifferentiated state can be regulated by oxygen tension. In hypoxic culture conditions, MSCs tend to change their phenotype toward a migratory type, as compared to cells maintained in normoxic culture conditions [24].

Even though the molecular mechanisms through which hypoxia increases ROS are still debated, it has been suggested that hypoxia may turn the mitochondrial electron transport chain (ETC) toward the production of ROS, in particular at the level of complexes I, II, and III. It has now been clearly demonstrated that ROS generation associated with hypoxia increases the proliferation and survival of human ADSCs [25]. When a local injury occurs, ROS and endogenous factors such as chemokines are produced from damaged cells, inducing ADSC migration.

In this context, from a clinical perspective, increasing the production rate and regenerative potential of ADSCs would be of great interest. Several pathways added to the ROS signal have been suggested as mediators of hypoxic adaptation; the primary mediators may be the different hypoxia inducible factors (HIFs). HIFs, and in particular HIF-2 alpha, appear to regulate the signaling pathways that regulate stem cell self-renewal and multipotency in various stem cells, and in particular in CSCs [26] and ADSCs [27]. ADSCs can produce and secrete many microvesicles and exosomes, which inherit multiple functions of cells. Vesicles released by cells into the extracellular environment are collectively named extracellular vesicles (EVs) and are classified depending on their origin and size. Exosomes have been proposed as a new means of intercellular communication [28], and they are released from many cell types, including stem cells. Exosomes represent a well-characterized subtype of EVs. Exosomes, microvesicles, and apoptotic bodies are all small sized and composed by a bilayer lipid membrane, and they are difficult to differentiate by current techniques. However, exosomes, whose biogenesis and release have been previously described [29,30], range from 40 to 100 nm diameter and have an endocytic origin.

The interactions between several types of exosomes and their target cells are described in the literature. The contact and release of an exosome’s contents into recipient cells is mediated by receptor–ligand interactions and/or by the direct fusion of lipid membranes and endocytosis [31]. The molecular content of exosomes depends on the cell type and the functional state of the producing cell. Their biological content includes lipids, metabolites, and proteins such as heat shock proteins (HSP), but also nucleotide content such as DNA and RNA (mRNA, microRNAs: miRNAs), and long noncoding RNAs (lncRNAs)). In this context, miRNAs from exosomal origin could regulate metabolism in distant tissues, and they are regarded as a novel class of chemokines with a new cell–cell crosstalk mechanism [32]. Some exosomal miRNAs have also been suggested as new potential biomarkers in the development of cardiovascular diseases [33]. The characteristics of exosomes derived from ADSCs (ADSC-Exos) have been previously described [34,35].

EVs and exosomes released by several type of cells act as antioxidant “factors” in recipient cells: antioxidant enzymes secreted in response to inflammatory signals [36]. A study performed on osteoarthritic progression confirmed that the treatment of osteoarthritic chondrocytes with extracellular vesicles derived from ADSCs resulted in a reduced production of inflammatory mediators (TNFα, IL-6, PGE2, and nitric oxide (NO)) and increased production of the anti-inflammatory cytokine IL-10 [37]. Finally, exosomes from ADSCs mediate autocrine, paracrine, and endocrine effects that can be exploited in cytoprotective and regenerative therapies [38].

## 3. Oxidative Stress: Imbalance between Oxidants and Antioxidants

**Figure 1:**
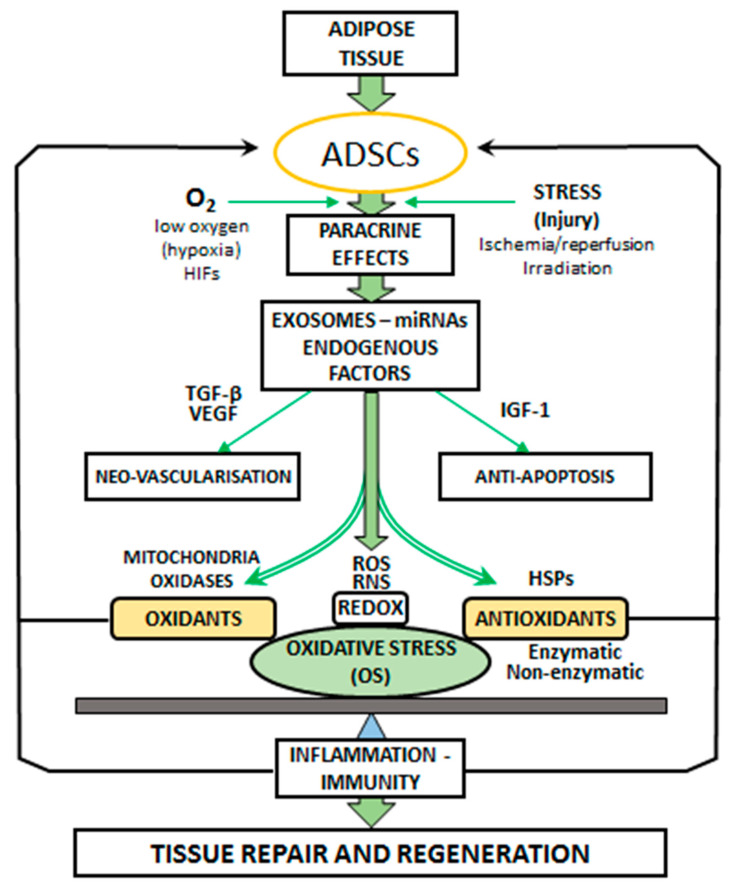
Paracrine effects of adipose-derived stem cells (ADSCs). ADSCs secrete vascular endothelial growth factor (VEGF), microRNAs (miRNAs), insulin-like growth factor-1 (IGF-1), and exosomes for promoting neovascularization and anti-apoptosis. ADSCs promote angiogenesis by producing vascular endothelial growth factor (VEGF) and transforming growth factor beta (TGF-β). Stress and local injury (ischemia/reperfusion, irradiation) induce ADSC activation. ADSCs exist in low oxygen conditions (hypoxia), and hypoxia-inducible factors (HIFs) regulate the signaling pathways. OS is the result of imbalance between the generation of oxidants: reactive oxygen species (ROS) and the antioxidant defense systems. The main cellular sources of ROS are mitochondria and oxidases. Antioxidants are represented by the enzymatic and non-enzymatic activities and HSP expression. The oxidation-reduction (REDOX) balance in the ROS and RNS (reactive nitrogen species: derived from nitric oxide: NO) mechanisms is involved in tissue repair and regeneration induced by ADSCs through the modification of OS levels. OS is recognized as a contributing factor in inflammation and immunity pathways.

The term ‘‘oxidative stress’’ (OS) is used to designate a number of biochemical, physiological, and pathophysiological situations. OS is a condition produced by the imbalance between oxidants and antioxidants in the biological systems. The imbalance occurs as a result of the excess level of active free radicals or inadequate functioning of the antioxidant system (Figure 1).

The term “radical” (or “free radical”) designated molecules or ions with one unpaired electron. Free radicals are highly reactive chemical species. ROS are produced as intermediates in redox reactions, leading from O_2_ to H_2_O. ROS and reactive nitrogen species (RNS) are the most important class of radical species generated in living systems. ROS and RNS, which are continuously released from normal cellular metabolism, play an ambivalent role with deleterious and beneficial effects [39,40].

Among the ROS produced in endothelial cells, the principal sources of superoxide include the nicotinamide dinucleotide phosphate (NADPH) oxidases (Nox), the xanthine oxidase, mitochondria and, in certain circumstances, the endothelial NO synthases [41,42]. Nox(s) were originally characterized in the phagocytes, which utilize a Nox-generated burst of superoxide anion (O_2_^•-^)s a mechanism to protect against pathogens and during aging [43]. Nox isoforms Nox2 and Nox4 have been identified in several different tissues. Nox4 is expressed at high levels both in white and brown pre-adipocytes, and differentiation into adipocytes results in a reduction of Nox4 mRNA content [44].

Some oxygen-derived radicals are extremely reactive with a short half-life. For example, ^•^OH can survive for 10^−10^ sec in biological systems. The life span of some other radicals is also short, but it depends on the environmental medium. In a hydrophilic environment, O_2_^•-^ is a reducing agent capable of reducing ferric (Fe ^3+^) ions to ferrous (Fe ^2+^) ions. Increased ROS production was associated with altered mitochondrial morphology. The hydrogen peroxide molecule (H_2_O_2_) produced by mitochondria does not contain an unpaired electron and thus is not a radical species but an oxidative agent [45]. H_2_O_2_ is a metabolite operative in redox sensing and signaling regulation.

Higher levels of O_2_ within mitochondria as a result of impaired oxidative metabolism may induce greater lipid peroxidation, activating signaling events that further worsen disease severity. In addition to the electron transport system, mitochondria also generate H_2_O_2_ from monoamine oxidase (MAO) that is bound to the outer membrane [46].

Cell exposure to ROS has resulted in the development of defense strategies through endogenous antioxidants. An antioxidant agent is a substance that limits or inhibits the substrate oxidation at very low concentrations compared to the oxidizable substrate. Defense mechanisms against ROS-induced toxicity include (1) preventive mechanisms, (2) repair mechanisms, and (3) antioxidant defenses. Enzymatic antioxidant defenses include superoxide dismutases (SODs), glutathione peroxidases (GPx), catalases (CAT), and other enzymes such as peroxiredoxines or thioredoxine. Ascorbic acid (vitamin C), tocopherol (vitamin E), and other direct antioxidants such as glutathione (GSH), folic acid, lipoic acid, thiols, or indirect antioxidants (chelate redox metals, pharmacological drugs) are non-enzymatic antioxidants. To protect the cellular structures from the effects of ROS, the antioxidants need to be located close to free radical production sites [47,48]. The organism must continuously challenge and control the presence of both pro-oxidants and antioxidants. Circulating OS can be quantified as the redox state of plasma [49].

## 4. Relationship between the Functions of ADSC and Oxidative Processes

The main questions concerning the crosstalk between ADSCs and OS are:

(1) Do ROS implicated in OS stimulate or limit ADSC activity?
(2) Are ADSC cells implicated in antioxidant processes?

### 4.1. Oxidative Stress during Ischemia/Reperfusion and Protection Induced by ADSCs

Ischemia–reperfusion (I/R) injury develops when the blood supply to an organ is disrupted and subsequently restored, and this process occurs in many conditions including heart attack and stroke. Substantial evidence supports the active involvement of free radicals in the functional alterations of an organ (such as the heart) after I/R. Many studies have provided direct evidence of a free radical burst starting in the first minutes of reperfusion. In conditions where the cellular antioxidative defense system is overwhelmed, the primary radical species may induce radical chain reactions leading to the appearance of secondary radical species [40]. ROS, as noted above, can serve as signaling molecules. Low levels of oxidants can induce cardioprotection, for example in preconditioning therapies, whereas high levels of ROS are deleterious and lead to cell death. Several research projects have aimed to reduce ROS levels by activating antioxidant defense systems in an attempt to counter the effect of ROS on viable cellular structures. ROS play an important role in the development of I/R injury. Antioxidant therapies limit the deleterious effects of ROS, but they are unable to prevent organ disorders. Larger studies testing antioxidant supplementation for the prevention of I/R events reported conflicting results [50].

Different protective or adaptive responses, mainly HIF pathway activation, have been observed during I/R, protecting cells from the consequences of oxygen deprivation by triggering metabolism programs [51]. In addition, the antioxidant transcription factor Nrf2 (nuclear factor erythroid 2-related factor 2) appears to be fundamental in mediating adaptive mechanisms for OS protecting the heart against I/R injury [52]. New therapeutic strategies have been suggested as approaches to enhance ischemia tolerance. In this field, ADSC-Exos are a novel cell-free therapy. For example, the exosomes seem to have an anti-apoptotic effect on cardiomyocytes through the Wingless-int (Wnt)/B-catenin pathway, thus protecting the myocardium against I/R injury [53]. Recent data have demonstrated that ADSC-Exos protect cardiomyocytes from OS. In order to determine the effects of exosomes on OS-induced apoptosis in cardiomyocytes, mouse cardiomyocyte (MCM) cells were co-cultured with ADSCs, and/or ADSC exosomes for 24 h. Then, co-cultures were treated using 0 or 200 μM H_2_O_2_. The number of apoptotic cells was significantly increased in H_2_O_2_-treated MCM cells, while co-culture with ADSC exosomes significantly suppressed H_2_O_2_-induced apoptosis [54].

Some studies have demonstrated that ADSC exosomes protect the kidney from I/R injury (kI/R), while others have shown that acute kI/R was associated with a significant inflammatory response and increased generation of ROS, causing damage to the kidneys [55,56].

In one experimental approach involving rats, ADSCs were administered via the tail vein following kI/R, and serum creatinine levels and creatinine clearance were assessed as markers of kidney function. OS was also determined through malondialdehyde (MDA) serum level measurements. Serum creatinine and serum and tissue MDA were significantly increased, and creatinine clearance was reduced significantly in response to kI/R. ADSCs confirmed their ability to reverse these changes by reducing the effect of OS in the serum and renal tissue [57]. These results are concordant with other studies demonstrating that ADSC therapy decreased kidney injury following I/R by limiting the OS level and the inflammatory response [58]. Histological and serum biochemical findings showed, respectively, that renal parenchymal damage and renal dysfunction were largely improved after ADSC treatment was administered. This suggests that ADSC treatment preserves renal function through various mechanisms: inhibition of inflammatory reactions, reduction of apoptosis, and OS during acute kI/R. Moreover, eNOS mRNA expression, an indicator of angiogenesis, was increased in animals after ADSC administration. In this process, it has been suggested that ADSC treatment may improve renal function after kI/R injury through an alternation in subcellular distribution of heme oxygenase-1 (HO-1) in cells under stress [59,60].

It is also important to remember that the protective function of exosomes has been attributed to HSPs. HSPs can restore redox balance, limit OS, prevent apoptosis pathway activation, and inhibit pro-inflammatory cytokines. Among the various HSPs, HSP70 and HSP27 can protect organs against I/R-associated injury [61,62]. HSP70 and HSP27 are secreted from cells by exosomes, and these proteins play a role in activating the adaptive and innate immune response [63,64].

Another study evaluated the anti-inflammatory and antioxidant effect of vitamin E treatment on porcine ADSCs against H_2_O_2_-induced OS. Gene expression analysis of vitamin-E-treated ADSCs showed downregulated expression of the genes associated with OS and apoptosis, including p53, BAX, NFκB, HIF1α, and VEGF-A genes. Vitamin E enhanced the survival and integrity of ADSC, which is associated with the reduction of factors contributing to inflammation and cell apoptosis [65]. As for the potential effects of ADSCs on the activity of genes involved in oxidative pathways, a recent study suggests fat-depot-specific differences in the cells derived from subcutaneous (SC) and visceral (VS) fat depots. Based on the expression of OS-inducing genes such as SODs, NOXs, NOS, CAT, and HO-1, it has been demonstrated that higher levels of ROS are found in VS-derived ADSCs and associated with cellular dysfunctions when compared with SC-derived ADSCs. VS-derived ADSCs show slower proliferation, which can be reversed by ascorbic acid supplementation [66]. The depot-specific differences in OS may be caused by several endogenous factors and environmental conditions. As previously stated, low oxygen tension is an important characteristic of the stem cell niche, and hypoxia provides a favorable environment for the properties of stem cells.

The cellular properties of ADSCs are modified by the metabolic environment. High levels of glucose result in stem cells apoptosis [67] and increase OS and autophagy in various cells. The process of autophagy creates interactions with proteins and signaling pathways that regulate cell properties, including phosphoinositide 3-kinase (PI3K)/Akt and mammalian target of rapamycin (mTOR) [68]. It has been recently reported that glucose-induced apoptosis in ADSCs in vitro was abolished by the upregulation of autophagy induced by rapamycin; in return, apoptosis levels were intensified by the inhibition of autophagy [69]. These results suggest that targeting ADSCs could be a new strategy for treating the complications of diabetes and improving tissue repair and regeneration.

It has been suggested that crosstalk between miRNA and OS involving the stem cells and exosomes occurs in various organs. During OS, NADPH is responsible for an upregulation of miRNA-21, miRNA 122, and miRNA-155, influencing forkhead box class O (FOXO): FOXO3a pathways and fibrosis [70]. Studies show that exosomes play an essential role in maintaining cell homeostasis during damage, and that circulating miRNA-122 is released from hepatocytes during liver injury as a defense mechanism [71]. MiRNA networks and OS are present in degenerative processes. OS affects the expression levels of various microRNAs and, conversely, microRNAs regulate many of the genes involved in an OS response. The Nrf2-antioxidant response element (ARE) pathway is an endogenous antioxidant system. OS induces the translocation of Nrf2 to the nucleus, activating gene expression, which encodes the proteins involved in the OS response. Various miRNAs released from exosomes can downregulate Nrf2 expression, influencing the modulation of OS response [72].

### 4.2. Oxidative Stress during Radiation and Protection Induced by ADSCs

ROS and RNS are mainly produced in cells by the radiolysis of water as the consequence of radiation. Given the fact that cells are primarily composed of water (likely more than 80%), ROS and RNS are the main origin of tissue damage [73].

There are several hypotheses to characterize the main targets of radiation injury in the skin, lung, and heart: free radical production, capillary lesions, mitochondrial alterations, and myofibrillar degeneration. The combination of radiotherapy and chemotherapy represents a major advance in the therapeutic management of cancer therapy. However, this association can cause severe side effects, including lesions on the vital organs, including the heart and lungs. Therefore, the challenge is to find ways to reduce the damage caused by the treatments or to avoid it altogether. Elevated levels of plasma and cardiac lipid peroxidation and plasma creatine kinase are early signs of cardiac peroxidative injury after radiation. Heart antioxidant depletion might lead to increasing peroxidase injury [74], while chronic fibrosis and acute pneumonia are the main types of radiation-induced damage in the lung. There is increasing evidence that miRNA is involved in various conditions, including radiation-induced lung damage, as reported in recent experimental studies [75]. Therefore, miRNAs may serve as biomarkers for the early stages of radiation-induced lung damage. Concerning protection against radiation-induced injury, many radioprotective drugs have been developed, and the role of ADSCs has recently been established. ADSCs have been shown to protect the skin and soft tissues in clinical radiotherapy injury and in animal experiments (mouse models of chronic radiotherapy injury). ADSCs are thought to play a supportive role in adipogenesis and angiogenesis in association with a modulation of inflammation and immunity [76].

During external radiotherapy, wound healing is heavily compromised by endothelial damage. Endothelial cells, which play a pivotal in wound healing, are highly affected and impacted by radiation. In wound healing, the concept of therapeutic angiogenesis has become widely accepted. It is now thought that ADSCs are able to migrate and differentiate into endothelial cells. One hypothesis is that ADSCs compensate for endothelial dysfunction after radiation by balancing the expression of soluble adhesion molecules. In an interesting in vitro study [77], human dermal microvascular endothelial cells (HDMEC) and human ADSCs were cultured in a co-culture setting and subjected to radiation. Assessed in the co-culture supernatant, interleukin-6 (IL-6), vascular cell adhesion molecule-1 (VCAM-1), intercellular adhesion molecule-1 (ICAM-1) and basic-fibroblast growth factor (b-FGF) levels were not as affected by the external radiation as HDMEC. The protective properties of ADSCs are associated with the reduction in pro-inflammatory cytokine release and the levels of vascular adhesion molecules [78].

The antioxidant effects of ADSCs were also demonstrated in dermal fibroblasts and epidermal keratinocytes after OS was induced with hydroperoxide or UVB [23]. The mechanism of action of the protective role of ADSCs against oxidative skin damage has been characterized. The protective mechanism observed after treatment with ADSC was an increase in the activity of antioxidant enzymes such as SODs and GPx in the dermal fibroblasts. Several reports have suggested that the wound-healing and antioxidant effect of ADSCs provide a potential approach to skin repair and regeneration [23,79,80].

The protective effect of ADSCs has also been studied in vitro and in vivo in degenerative diseases of the retina associated with OS. The retina is highly susceptible to OS through the production of ROS, which induces retinal pigment epithelium (RPE) cell death, leading to photoreceptor degradation. This damage contributes to retinal degeneration in conditions such as diabetes [47,81]. Other studies confirmed that ADSCs were able to prevent primary human RPE cell death caused by OS induced with H_2_O_2_. ADSC-treated mice showed a preservation of nuclear layers, RPE, and photoreceptors in comparison with control mice [82]. These data demonstrate the beneficial properties of ADSCs and provide a basis for therapeutic approaches to diseases of the retina.

Finally, similar to other stem cells, ADSCs are resistant to OS and play a key role in protecting cells from oxidative damage. ADSCs are thought to play a role in modulating inflammation and immunity, but the cellular mechanisms have not yet been entirely identified.

## 5. Concluding Remarks

In conclusion, ADSC therapy has demonstrated beneficial effects, suggesting that secreted factors may provide protection. There is evidence that ADSCs secrete a number of cytokines, growth factors, and antioxidant factors into their microenvironment, thus regulating intracellular signaling pathways in neighboring cells. Pharmacological treatment of ADSCs with drugs such as rosuvastatin increases the efficacy of their paracrine functions [83]. The beneficial effect may be related to the antioxidant properties demonstrated with this statin. For instance, experimental and clinical studies have shown that rosuvastatin decreased the levels of OS markers in vivo [84,85]. Therefore, adjuvant therapy with rosuvastatin is a novel strategy for promoting the clinical efficacy of ADSCs. Nonetheless, further research is needed to understand the immunomodulatory and endocrine properties of ADSCs that could be used for the treatment of conditions linked to chronic inflammation. Further investigations are also required to clarify the therapeutic properties of ADSCs that could be used to benefit patients suffering from cardiovascular and muscular diseases. Overall, ADSCs are a promising source of future cell-based therapies for human disease and tissue regeneration [80]. Regenerative therapies are in high demand as a new treatment strategy, and several cell-based strategies are currently being explored for cardiac regeneration.

## Abbreviations

CSCs	cancer stem cells
DFX	deferoxamine
ECs	endothelial cells
ESCs	embryonic stem cells
ETC	electron transport chain
EVs	extracellular vesicles
FGF	fibroblast growth factor
FOXO	forkhead box class O
GPx	glutathione peroxidase
GSH	glutathione
HDMEC	human dermal microvascular endothelial cells
HIF	hypoxia inducible factor
HO-1	heme oxygenase-1
HSCs	hematopoietic stem cells
HSP	heat shock proteins
ICAM-1	intercellular adhesion molecule-1
IGF	insulin-like growth factor
IL	interleukin
lncRNAs	long noncoding RNAs
iPSCs	induced pluripotent stem cells
I/R	ischemia/reperfusion
KI/R	kidney I/R
MAO	monoamine oxidase
MCM	mouse cardiomyocyte
MiRNA	microRNA
MSCs	mesenchymal stem/stromal cells
NO	nitric oxide
Nox	nicotinamide dinucleotide phosphate (NADPH) oxidases
Nrf2	nuclear factor erythroid 2-related factor 2
OS	oxidative stress
PSC	pluripotent stem cells
RNS	reactive nitrogen species
ROS	reactive oxygen species
RPE	retinal pigment epithelium
SC	subcutaneous
SOD	superoxide dismutase
TGF-β	transforming growth factor beta
VCAM-1	vascular cell adhesion molecule-1
VEGF	vascular endothelial growth factor
VS	visceral
